# Interleukin-34 overexpression mediated through tumor necrosis factor-alpha reflects severity of synovitis in knee osteoarthritis

**DOI:** 10.1038/s41598-020-64932-2

**Published:** 2020-05-14

**Authors:** Wanvisa Udomsinprasert, Artit Jinawath, Nipaporn Teerawattanapong, Sittisak Honsawek

**Affiliations:** 1 Department of Biochemistry, Faculty of Pharmacy, Mahidol University, Bangkok, 10400 Thailand; 2 Department of Pathology, Faculty of Medicine, Ramathibodi Hospital, Mahidol University, Bangkok, 10400 Thailand; 3 Department of Biochemistry, Osteoarthritis and Musculoskeleton Research Unit, Faculty of Medicine, Chulalongkorn University, King Chulalongkorn Memorial Hospital, Thai Red Cross Society, Bangkok, 10330 Thailand; 4 Research Division, Department of Research and Development, Faculty of Medicine, Siriraj Hospital, Mahidol University, Bangkok, 10700 Thailand

**Keywords:** Biochemistry, Molecular biology, Biomarkers, Medical research, Rheumatology

## Abstract

This study aimed to investigate whether interleukin-34 (*IL-34*) mRNA expression is aberrant and modulated by tumor necrosis factor-alpha (TNF-α) in knee osteoarthritis (OA) fibroblast-like synoviocytes (FLS) and determine associations of IL-34 mRNA and protein in the systemic and local joint environments with severity of knee OA synovitis. Transcriptional and translational IL-34 levels in FLS, synovium, synovial fluid, and plasma of knee OA were determined using real-time polymerase chain reaction, immunohistochemistry, and enzyme-linked immunosorbent assay. Relative mRNA expressions of NF-κB signaling molecules were further measured. In knee OA FLS stimulated with TNF-α, *IL-34* mRNA expression was significantly up-regulated in a time-dependent manner. In knee OA synovium with severe synovitis, increased *IL-34* mRNA expression was directly associated with *IL-6*, *IκB*, *NF-κB*, and *MMP-13*, in addition to knee OA FLS. Immunostaining score of IL-34 was considerably greater in knee OA synovium with severe synovitis than that in those with mild and no synovitis. Increments in joint fluid and plasma IL-34 levels in knee OA patients with severe synovitis were closely related to its mRNA and protein expressions in knee OA synovium. Transcriptional and translational expressions of IL-34 were positively correlated with synovitis severity. Collectively, IL-34 overexpression would reflect synovitis severity in knee OA.

## Introduction

Knee osteoarthritis (OA) is a degenerative joint disease mainly afflicting the elderly where the patients will develop severe pain towards disability and eventually require total knee replacement (TKR), which creates a huge and continuously growing burden on individuals and society^1^. Although knee OA is appreciated to be extraordinarily complex with a poorly defined pathogenesis, inflammation of the synovium (also known as synovitis) has been recognized as a prominently pathologic event driving the progressive joint degeneration in knee OA^2^. Mechanistically, products of degraded cartilage fragments released into the synovial cavity are likely to initiate synovitis in knee OA, through which synovial fibroblasts, macrophages, and chondrocytes secrete inflammatory cytokines as critical contributors to synovitis^3,4^. These cytokines aggrandize the production of proteolytic enzymes responsible for cartilage breakdown^5^. It is noteworthy that identifying early changes in the synovium could allow for a larger window of opportunity during which alleviating action could be taken before the onset of irreversible changes and aggravating disabilities. In this context, the molecules known to play a possible part in inflammatory process have great potential to become diagnostic biomarkers for the progressive synovitis of knee OA.

As an alternative ligand for colony-stimulating factor-1 receptor (CSF-1R) functionally involved in the differentiation and proliferation of myeloid lineage cells responsible for inflammation and bone erosion, interleukin-34 (IL-34) has been hypothesized to be a molecular predictor for the development and progression of knee OA synovitis. In regard to its effect on inflammatory response, an experimental study unveiled that IL-34 knock-down mice attenuate the severity of inflammatory arthritis^6^. More specifically, it has been demonstrated that the biological action of IL-34 involved in inflammation is mediated through pro-inflammatory cytokines including tumor necrosis factor-alpha (TNF-α) and IL-1β in fibroblast-like synoviocytes (FLS) isolated from rheumatoid arthritis (RA) patients^7,8^. These pro-inflammatory cytokines have been shown to urge the secretion and expression of IL-34 through the activation of nuclear factor kappa B (NF-κB) and c-Jun N-terminal kinase (JNK) signaling pathways^8,9^. Indeed, overexpressions of IL-34 mRNA and protein in the inflamed synovium of RA patients were reportedly associated with the severity of synovitis^7,8,10^. Besides altered mRNA expression of *IL-34*, accumulating data derived from clinical studies uncovered an increase in the systemic and local levels of IL-34 in patients with inflammatory arthritis^11^. The above-mentioned findings led us to consider the hypotheses that IL-34 would be a downstream effector of pro-inflammatory cytokines, which are pivotal mediators of synovitis, and could have potential as a biochemical indicator for synovitis in knee OA.

Although the pathological role of IL-34 substituted for CSF in osteoclastogenesis-induced bone erosion and its significant involvement in RA synovitis have been clearly understood and thoroughly explored, the expression and secretion of IL-34 in the synovium and knee OA FLS have received less attention. Accordingly, the present study aimed to investigate transcriptional and translational expressions of IL-34 in knee OA synovium and determine the possible associations of its mRNA and protein expressions with the degree of synovitis. Whether aberrant mRNA expression of *IL-34* in knee OA FLS is regulated by a pro-inflammatory cytokine like TNF-α and associated with gene expressions of *NF-κB* and its signaling molecules was further determined.

## Results

### Clinical characteristics of study subjects

Baseline demographic and clinical characteristics of study participants are summarized in Table 1. There were no significant differences in mean age, gender ratio, or body mass index (BMI) among knee OA with different degrees of synovitis.

**Table 1:** Baseline characteristics of knee OA patients among different groups.

Variables	Knee OA patients				*P*-value
	Total	No synovitis	Mild synovitis	Severe synovitis	
Number	50	16	19	15	
Age (years)	72.2 ± 8.0	74.0 ± 6.3	70.8 ± 8.6	73.3 ± 8.2	0.57
Gender (female/male)	38/12	11/5	15/4	12/3	0.84
BMI (kg/m^2^)	24.9 ± 3.5	24.0 ± 3.4	25.0 ± 2.8	25.9 ± 4.7	0.53

Abbreviations: BMI, body mass index; OA, osteoarthritis.

### Increased *IL-34* mRNA expression modulated by TNF-α in knee OA FLS

**Figure 1:**
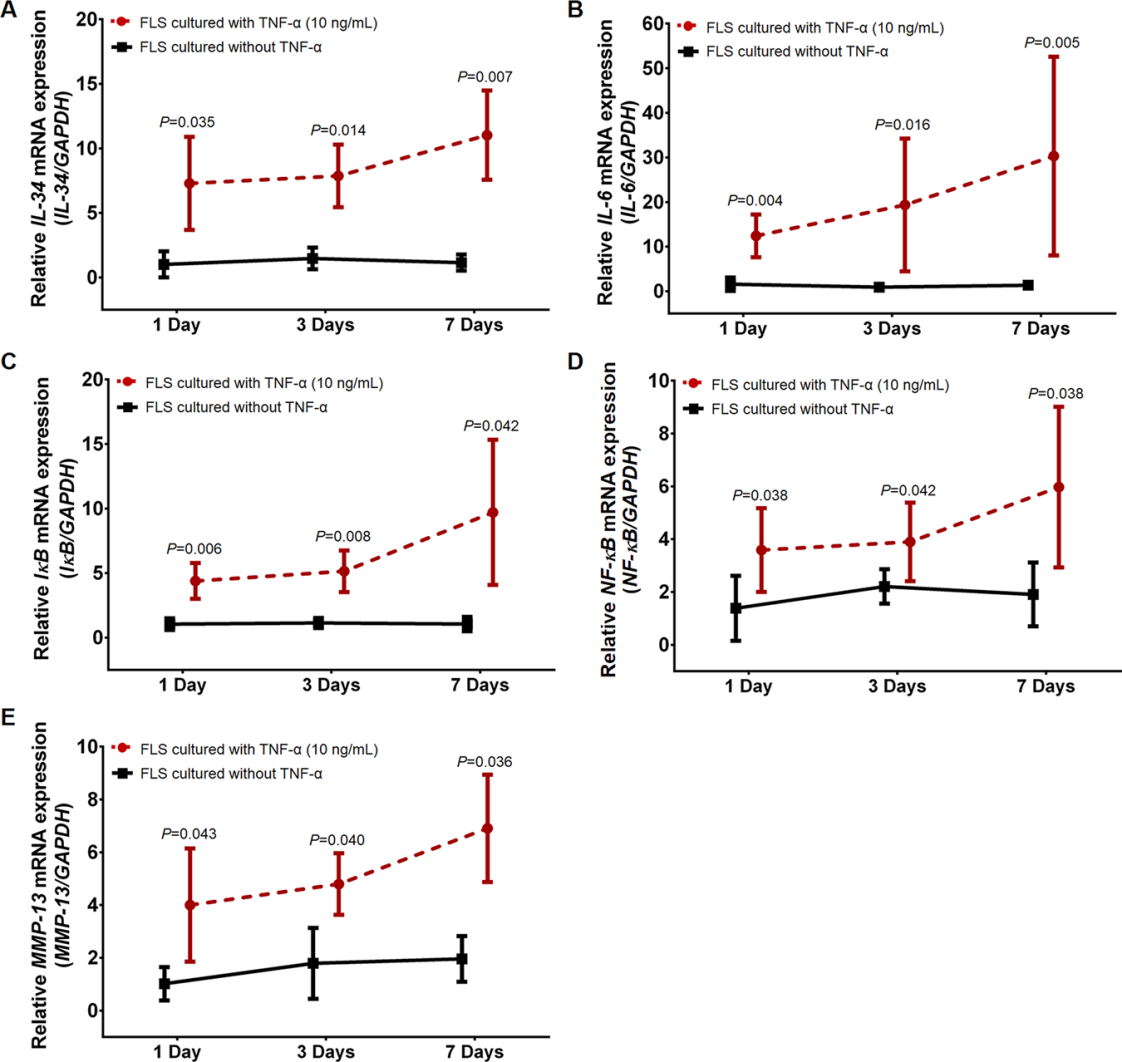
Relative mRNA expressions of *IL-34* and signaling molecules relevant to NF-κB pathway in knee OA FLS treated with and without TNF-α at various time-points. (**A**) *IL-34*. (**B**) *IL-6*. (**C**) *IκB*. (**D**) *NF-κB*. (**E**) *MMP-13*.

As TNF-α is a key molecule known to regulate inflammatory response in the synovium via enhancing the production of inflammatory mediators mediated through the activation of NF-κB signaling pathway, we firstly explored whether TNF-α increases mRNA expressions of *IL-34* and signaling molecules relevant to NF-κB pathway in FLS isolated from 4 knee OA synovium (average age 76.5 ± 7.2 years, 2 women and 2 men). Relative mRNA expressions of *IL-34*, *IL-6, IκB*, *NF-κB*, and matrix metalloproteinase-13 (*MMP-13)* in knee OA FLS stimulated with or without 10 ng/mL TNF-α for 1, 3, and 7 days were determined by real-time polymerase chain reaction (PCR). Compared with untreated knee OA FLS, relative *IL-34* mRNA expression was significantly escalated in a time-dependent manner in knee OA FLS stimulated with TNF-α (*P* = 0.035, *P* = 0.014, *P* = 0.007, respectively) (Fig. 1A). Consistent with this finding, gene expression analysis of signaling molecules involved in NF-κB pathway showed a time-dependent manner of markedly up-regulated mRNA expressions of *IL-6* (*P* = 0.004, *P* = 0.016, *P* = 0.005, respectively)*, IκB* (*P* = 0.006; *P* = 0.008; *P* = 0.042, respectively), *NF-κB* (*P* = 0.038; *P* = 0.042; *P* = 0.038, respectively), and *MMP-13* (*P* = 0.043; *P* = 0.040; *P* = 0.036, respectively) in knee OA FLS with TNF-α stimulation, when compared with those without TNF-α stimulation (Fig. 1B–E).

### Up-regulated mRNA expression of *IL-34* in knee OA synovitis

**Figure 2:**
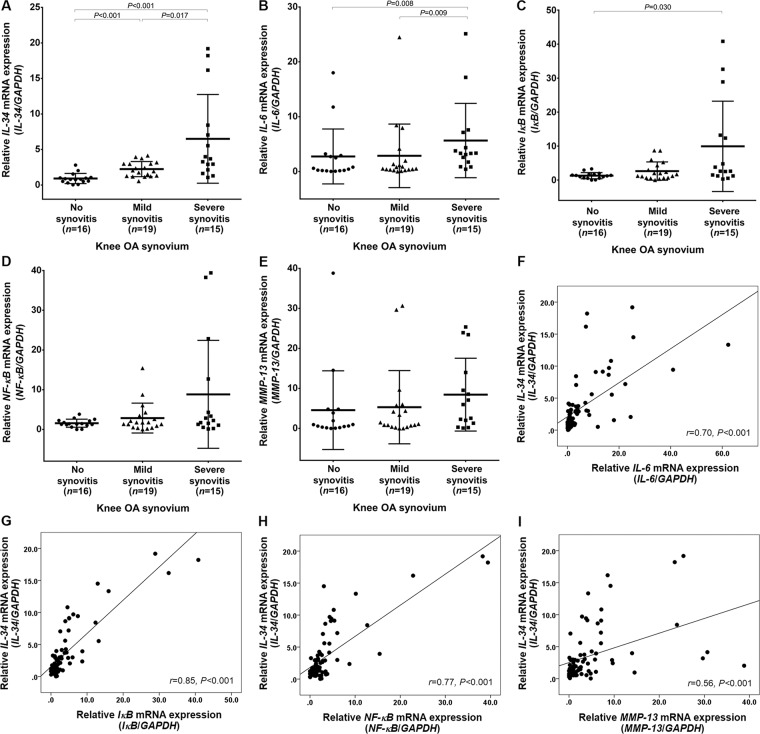
Relative mRNA expressions of *IL-34* and signaling molecules involved in NF-κB pathway in knee OA synovium. (**A**) *IL-34* in knee OA synovium with and without synovitis. (**B**) *IL-6* in knee OA synovium with and without synovitis. (**C**) *IκB* in knee OA synovium with and without synovitis. (**D**) *NF-κB* in knee OA synovium with and without synovitis. (**E**) *MMP-13* in knee OA synovium with and without synovitis. (**F**) Association between *IL-34* and *IL-6*. (**G**) Association between *IL-34* and *IκB*. (**H**) Association between *IL-34* and *NF-κB*. (**I**) Association between *IL-34* and *MMP-13*.

Due to up-regulation of *IL-34* mRNA expression in knee OA FLS stimulated with TNF-α, we determined relative mRNA expressions of *IL-34* in synovial biopsies obtained from 50 knee OA patients. According to the severity of synovitis assessed by histopathological grading system with hematoxylin and eosin (H&E) staining, synovial tissues of knee OA were classified into no synovitis (0–1 point, *n* = 16), mild synovitis (low-grade: 2–4 points, *n* = 19), and severe synovitis groups (high-grade: 5–9 points, *n* = 15). Quantitative real-time PCR showed that relative *IL-34* mRNA expression was significantly higher in knee OA synovium with severe synovitis than that in those with mild synovitis (*P* = 0.017) and no synovitis (*P* < 0.001). Correspondingly, knee OA synovium with mild synovitis remained remarkably more pronounced *IL-34* mRNA expression, as compared with those without synovitis (*P* < 0.001) (Fig. 2A).

To investigate whether up-regulated *IL-34* mRNA expression is associated with signaling molecules relevant to inflammatory process in knee OA synovium, we further examined mRNA expressions of *IL-6, IκB, NF-κB*, and *MMP-13*. As illustrated in Fig. 2B,C, relative *IL-6* mRNA expression in knee OA synovium with severe synovitis was significantly greater than that in those with mild synovitis (*P* = 0.009) and no synovitis (*P* = 0.008), and a marked up-regulation of *IκB* mRNA expression was observed in knee OA synovium with severe synovitis compared with those without synovitis (*P* = 0.030). Alternatively, *NF-κB* and *MMP-13* mRNA expressions were found to be higher in knee OA synovium with severe synovitis than that in those with mild and no synovitis, but these differences were not statistically significant (Fig. 2D,E).

Interestingly, subsequent analysis revealed that *IL-34* mRNA expression was positively associated with mRNA expressions of *IL-6* (*r* = 0.70, *P* < 0.001)*, IκB* (*r* = 0.85, *P* < 0.001)*, NF-κB* (*r* = 0.77, *P* < 0.001), and *MMP-13* (*r* = 0.56, *P* < 0.001) in both FLS and the synovium of knee OA (Fig. 2F–I).

### Overexpression of IL-34 protein in knee OA synovitis

**Figure 3:**
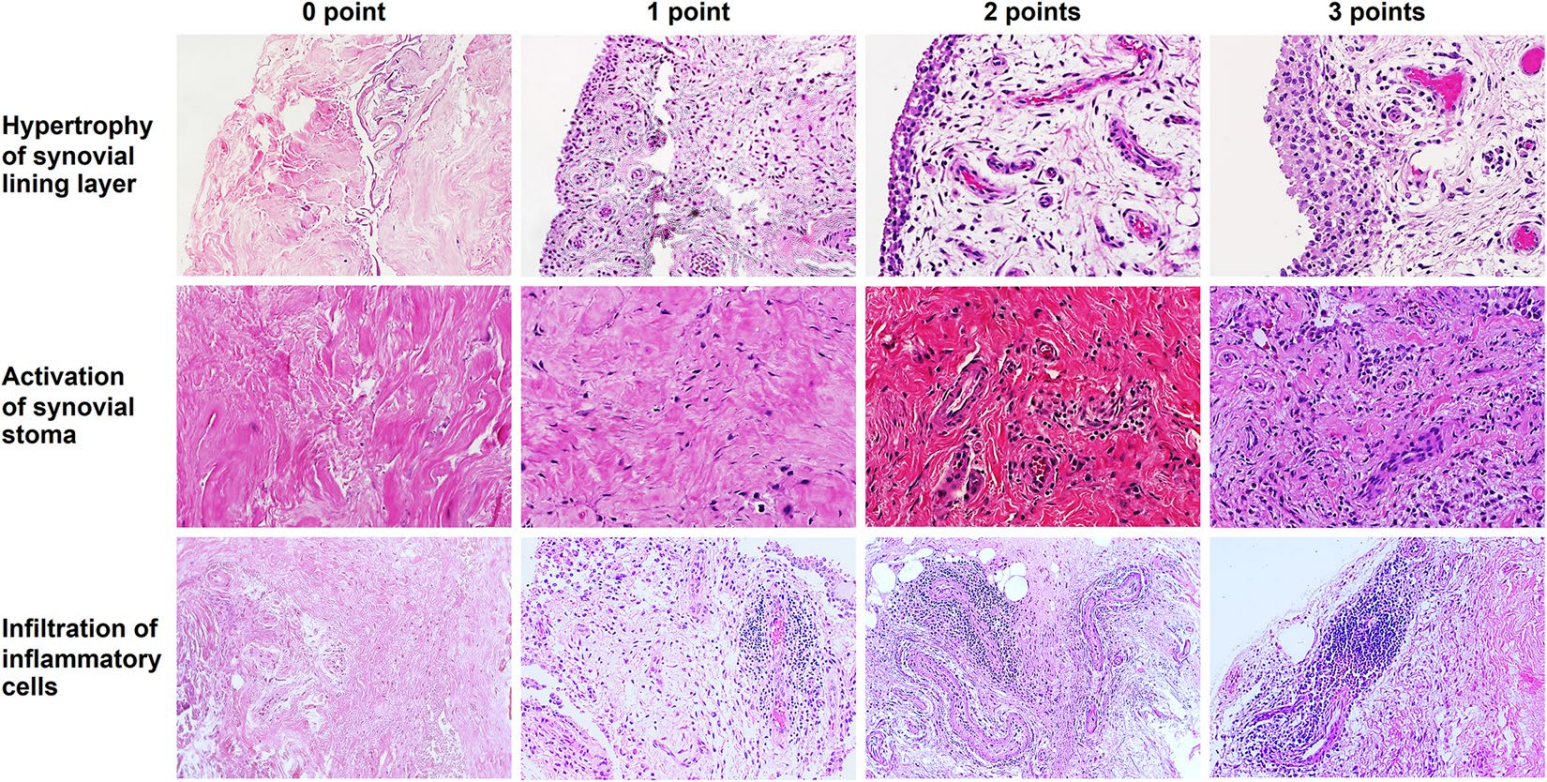
Histopathological staining in knee OA synovium. Knee OA with synovitis manifested morphological changes in the synovium including hypertrophy of synovial lining layer, activation of synovial stoma, and infiltration of inflammatory cells.

The severity of knee OA synovitis was assessed by H&E staining, according to three histological features of synovial membrane including enlargement of synovial lining cell layer, activation of synovial stroma, and infiltration of inflammatory cells, as illustrated in Fig. 3. Of 50 knee OA synovial biopsies, 15 (30%) were defined as high-grade synovitis (5–9 points), 19 (38%) were identified as low-grade synovitis (2–4 points), and 16 (32%) were scored as no synovitis (0–1 point), with regard to the histopathological synovitis score. In high-grade synovitis, H&E staining depicted enlarged widening of the surface cell layer, increased cell density of the synovial stroma, and elevated density of inflammatory leukocytic infiltration, compared with low-grade knee OA synovitis. On the other hand, the absences of synovial hypertrophy, activation of synovial stroma, and infiltration of inflammatory cells were observed in knee OA synovium without synovitis.

**Figure 4:**
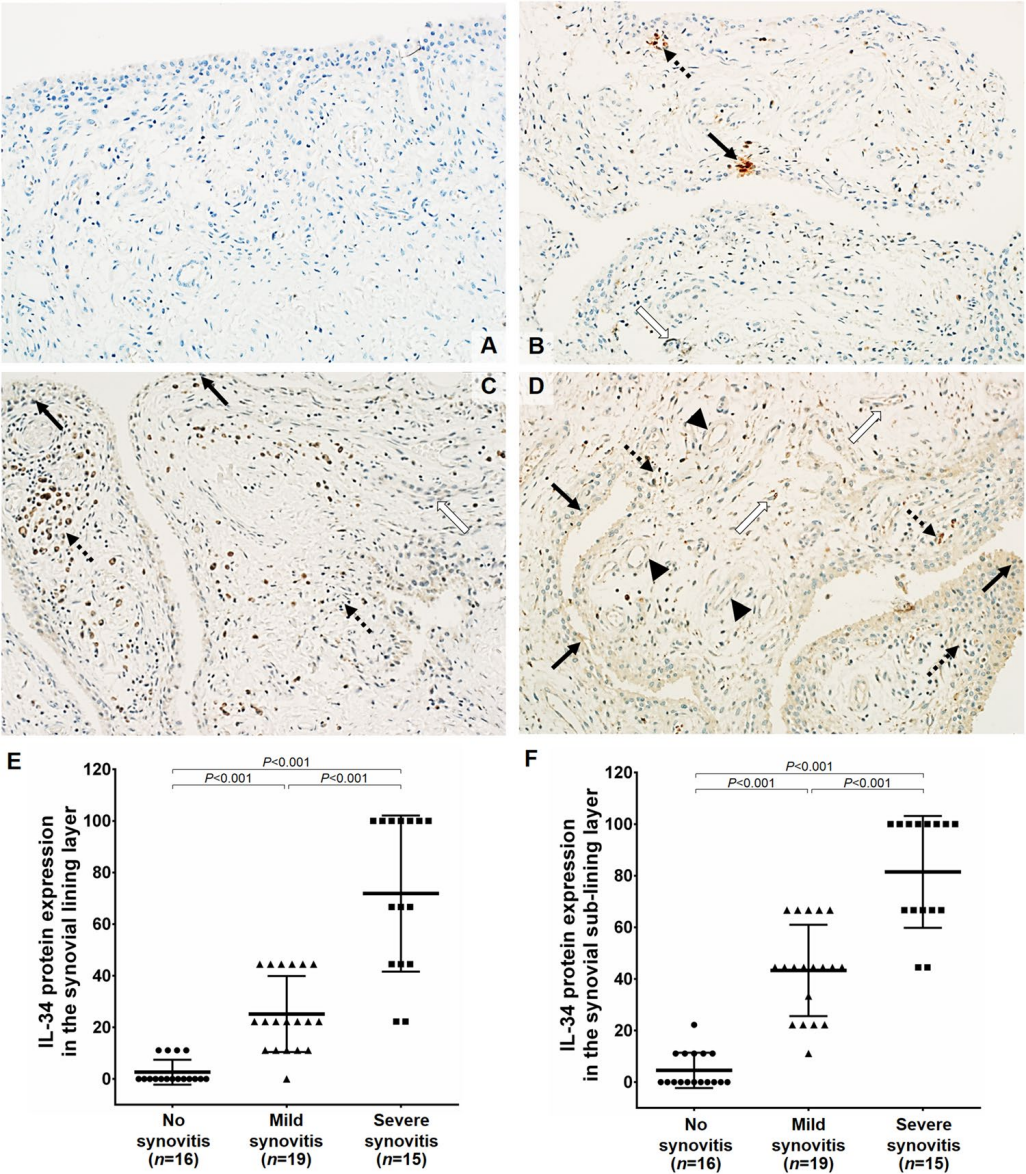
Immunohistochemical staining for IL-34 protein expression. (**A**) 0 = no expression of IL-34. (**B**) 1 = mild expression of IL-34. (**C**) 2 = moderate expression of IL-34. (**D**) 3 = strong expression of IL-34. (**E**) Immunoreactive scores of IL-34 in the synovial lining layer of knee OA. (**F**) Immunoreactive scores of IL-34 in the synovial sub-lining layer of knee OA.

In parallel with the histopathological staining of synovitis, immunohistochemical evaluation for IL-34 protein expression was performed in the synovial tissues of knee OA patients (*n* = 50). Representative immunohistochemical findings of IL-34 are demonstrated in Fig. 4. In low- and high-grade synovitis of knee OA, IL-34 expression was detectable in the synovial lining cell layer, predominantly in synoviocytes (arrows) as well as inflammatory cells (dotted arrows) and the synovial sub-lining cell layer, notably in epithelial cells (head arrows) and fibroblasts (white arrows) (Fig. 4B–D). In contrast to this, IL-34 was scarcely expressed in knee OA synovium without synovitis, being demonstrated as faint cytoplasmic staining (Fig. 4A).

In order to estimate expression levels of IL-34 protein in knee OA synovium among different synovitis groups, the staining intensity and percentage of IL-34 positive cells were assessed by a visual scoring method represented as a total score of staining on histological sections of the synovium. Immunoreactive analysis unveiled that IL-34 protein expression in the synovial lining layer of high-grade synovitis was remarkably greater than that in the synovial lining layer of low-grade synovitis (*P* < 0.001) and no synovitis (*P* < 0.001), as depicted in Fig. 4E. In the synovial sub-lining layer of knee OA, IL-34 protein expression was significantly higher in knee OA synovium with high-grade synovitis than in those with low-grade synovitis (*P* < 0.001) and without synovitis (*P* < 0.001) (Fig. 4F).

### Elevated synovial fluid and plasma IL-34 levels in knee OA synovitis

**Figure 5:**
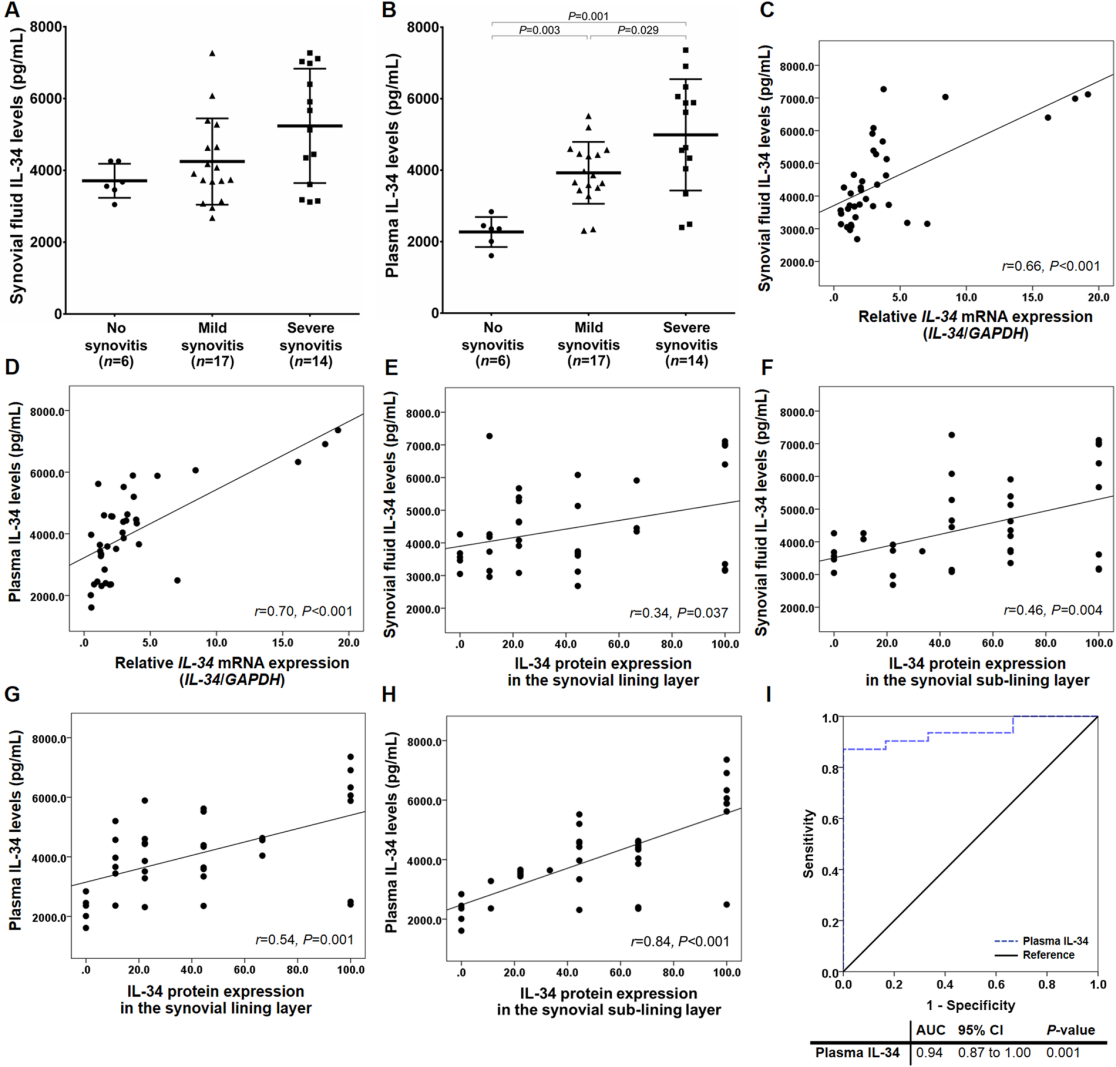
Synovial fluid and plasma IL-34 levels in knee OA patients. (**A**) Synovial fluid IL-34 in knee OA patients among synovitis subgroups. (**B**) Plasma IL-34 in knee OA patients among synovitis subgroups. (**C**) Association between synovial fluid IL-34 and its mRNA expression in knee OA synovium. (**D**) Association between plasma IL-34 and its mRNA expression in knee OA synovium. (**E**) Association between synovial fluid IL-34 and its protein expression in the synovial lining layer of knee OA. (**F**) Association between synovial fluid IL-34 and its protein expression in the synovial sub-lining layer of knee OA. (**G**) Association between plasma IL-34 and its protein expression in the synovial lining layer of knee OA. (**H**) Association between plasma IL-34 and its protein expression in the synovial sub-lining layer of knee OA. (**I**) ROC curve revealing clinical usefulness of plasma IL-34 as a novel biomarker for knee OA synovitis.

Apart from its mRNA and protein expressions in knee OA synovium, we additionally measured IL-34 protein levels in available synovial fluid and plasma samples from 37 knee OA patients using ELISA. As demonstrated in Fig. 5A, there was no significant difference in synovial fluid IL-34 levels among the patients with different groups. On the other hand, knee OA patients with severe synovitis had considerably increased plasma IL-34 levels compared with those with mild synovitis (*P* = 0.029) and without synovitis (*P* = 0.001), and its plasma levels in the patients with mild synovitis were significantly higher than that in those with no synovitis (*P* = 0.003) (Fig. 5B).

In stratified analysis, both synovial fluid and plasma IL-34 levels were found to be directly correlated with its mRNA expression in knee OA synovium (*r* = 0.66, *P* < 0.001; *r* = 0.70, *P* < 0.001, respectively), consistent with their correlations with protein expression in the synovial lining and sub-lining layers of knee OA (*r* = 0.34, *P* = 0.037; *r* = 0.46, *P* = 0.004*; r* = 0.54, *P* = 0.001; *r* = 0.84, *P* < 0.001, respectively), as depicted in Fig. 5C–H.

### Plasma IL-34 as a non-invasive biomarker for knee OA synovitis

Due to lack of reliable and specific biomarkers for knee OA synovitis, the AUC of the ROC curve was constructed to determine the possibility whether plasma IL-34 could be employed as a useful biomarker for the developmental synovitis. Based on the ROC curve, the optimal cutoff value of plasma IL-34 as a non-invasive marker for distinguishing knee OA patients with synovitis from those without synovitis was projected to be 3,060 pg/mL, which yielded a sensitivity of 87.1%, a specificity of 100.0%, and an AUC of 0.94 (95% CI: 0.87 to 1.00; *P* = 0.001) (Fig. 5I).

### Positive correlations of IL-34 mRNA and protein expressions with the synovitis severity

Table 2 demonstrates the possible associations of IL-34 mRNA and protein expressions in the systemic and local joint environments with the degree of synovitis in knee OA. Spearman’s rho correlation analysis demonstrated that there was a significantly direct link between *IL-34* mRNA expression and the synovitis severity in knee OA synovium (*r* = 0.75, *P* < 0.001), concordant with the associations of IL-34 protein expression in both the synovial lining layer (*r* = 0.88, *P* < 0.001) and sub-lining layer (*r* = 0.92, *P* < 0.001) with the severity of synovitis. In joint fluid and plasma of knee OA, IL-34 levels were observed to be positively correlated with the degree of synovitis (*r* = 0.65, *P* < 0.001*; r* = 0.74, *P* < 0.001, respectively).

**Table 2:** Spearman’s correlation and multivariate linear regression analysis of synovitis estimates.

Variables	Degree of synovitis (0–9 points)			
	Spearman’s rho correlation		Linear regression^a^	
	Coefficient (*r*)	*P*-value	β coefficient (95% CI)	*P*-value
Age (years)	0.05	0.76	—	—
Gender (female/male)	−0.05	0.71	—	—
BMI (kg/m^2^)	0.18	0.33	—	—
*IL-34* mRNA expression	0.75	<0.001	0.399 (0.224 to 0.575)	<0.001
IL-34 protein expression in the synovial lining layer	0.88	<0.001	0.066 (0.053 to 0.079)	<0.001
IL-34 protein expression in the synovial sub-lining layer	0.92	<0.001	0.069 (0.054 to 0.083)	<0.001
Synovial fluid IL-34 (pg/mL)	0.65	<0.001	0.001 (0.000 to 0.002)	0.004
Plasma IL-34 (pg/mL)	0.74	<0.001	0.001 (0.000 to 0.002)	0.002

^a^The coefficient was adjusted for age, gender, and BMI.

Abbreviations: BMI, body mass index; IL-34, interleukin-34.

After adjusting for confounding factors including age, gender, and BMI, multivariate linear regression analysis showed that the degree of synovitis was independently associated with *IL-34* mRNA expression (β coefficient=0.399, 95% CI: 0.224 to 0.575, *P* < 0.001) and its protein expression in the synovial lining (β coefficient=0.066, 95% CI: 0.053 to 0.079, *P* < 0.001) and sub-lining layers (β coefficient=0.069, 95% CI: 0.054 to 0.083, *P* < 0.001), in addition to its synovial fluid and plasma levels (β coefficient=0.001, 95% CI: 0.000 to 0.002, *P* = 0.004; β coefficient=0.001, 95% CI: 0.000 to 0.002, *P* = 0.002, respectively).

## Discussion

It is well-known that pro-inflammatory cytokines including IL-1β, IL-6, and TNF-α possess the important roles in synovitis and cartilage degradation^12–15^, suggesting their pathological action in inflammatory arthritis. Through the activation of NF-κB signaling cascade, TNF-α reportedly magnifies the production of inflammatory mediators, matrix metalloproteinases (MMPs), and chemokines in FLS isolated from the inflamed synovium of RA, which in turn accelerates osteoclasts formation-induced bone loss^15–17^. As an alternative ligand for CSF-1R, IL-34 has been identified to be a novel regulatory mediator for the differentiation and function of osteoclasts^18,19^, thereby highlighting a possible link between IL-34 and TNF-α under the pathological inflammatory conditions. Supporting this, a number of experimental studies demonstrated that aberrant IL-34 expression in osteoblasts was regulated by TNF-α through the activation of NF-κB signaling pathway^20,21^. Besides these previous findings, a dose-dependent manner of increased *IL-34* mRNA expression has been reportedly induced by TNF-α-stimulated RA FLS, which was mediated through JNK and NF-κB activities^8^. Collectively, the above-mentioned findings led us to speculate that IL-34 could be a downstream effector of TNF-α and may exert its probable effects on synovial inflammation and cartilage breakdown in knee OA. Attesting these assumptions, our findings unveiled a time-dependent manner of up-regulated *IL-34* mRNA expression in knee OA FLS stimulated with TNF-α. In addition to this, relative mRNA expressions of signaling molecules implicated in NF-κB pathway including *IL-6*, *IκB*, *NF-κB*, and *MMP-13* were found to be significantly increased in knee OA FLS after TNF-α treatment, compared with untreated knee OA FLS. Apart from its mRNA expression in knee OA FLS, this study examined *IL-34* mRNA expression in knee OA synovium with regard to the severity of synovitis and revealed up-regulation of *IL-34* mRNA expression in severe synovitis of knee OA synovium, consistent with *IL-6* and *IκB* mRNA expression analyses demonstrating significant up-regulations of their mRNA expressions in severe synovitis of knee OA synovium. More importantly, the significantly positive associations of *IL-34* mRNA expression with mRNA expressions of *IL-6*, *IκB*, *NF-κB*, and *MMP-13* were observed in both knee OA FLS and synovium. These findings shed light on a possible link between IL-34 expression and NF-κB signaling pathway implicated in synovitis-persuaded cartilage breakdown. In parallel with increased *IL-34* mRNA expression in knee OA synovium, its protein overexpression localized in the synovial lining layers, especially in synoviocytes and inflammatory cells and the synovial sub-lining layers, predominantly in epithelial cells and fibroblasts was found in knee OA synovium with synovitis – notably those with severe synovitis. Our subsequent analysis further uncovered the positive associations of IL-34 mRNA and protein expressions with the severity of knee OA synovitis. These findings are in line with cumulative data from clinical studies, which noted overexpressions of IL-34 mRNA and protein in the inflamed synovium of RA and a close link between its expression and synovitis severity^8,9^. All above-mentioned findings provide further evidence supporting IL-34 significant involvement in synovial inflammation of patients with arthritis – especially knee OA. Given that transcriptional and translational overexpressions of IL-34 have been delineated in knee OA FLS treated with TNF-α and the inflamed synovium, whether its levels in the synovial fluid and circulation are associated with the severity of knee OA synovitis was determined in the current study. Our additional results depicted that plasma IL-34 levels were significantly increased in knee OA patients with severe synovitis compared with those with mild synovitis and without synovitis, whereas synovial fluid IL-34 levels tended to be greater in knee OA patients with severe synovits than that in those with mild and no synovitis. More precisely, its plasma and synovial fluid levels were observed to be positively related to the severity of synovitis. In support of increases in joint fluid and circulating IL-34 levels in knee OA patients, a recently published research denoted that serum IL-34 levels were considerably higher in knee OA patients than that in healthy controls, and synovial fluid IL-34 levels were considerably greater than that in paired serum samples of knee OA patients^22^. Besides altered joint fluid and circulating IL-34 levels in knee OA patients, previous studies showed that increments in synovial fluid and circulating IL-34 levels were directly correlated with clinical variables of RA patients^7,8,10,23^. From the aforementioned previous findings, it has been postulated that alterations in transcriptional and translational production of IL-34 would reflect the severity of synovitis in knee OA patients, and its circulating levels could emerge as a non-invasive biomarker for the progression of knee OA synovitis. To date, early diagnosis of knee OA and the ability to track its synovitis progression are challenging. In an attempt to achieve early detection of knee OA synovitis, this study is the first to demonstrate a clinical usefulness of plasma IL-34 as a potential biochemical marker for discriminating knee OA patients with synovitis from those without synovitis, in which ROC curve analysis showed the optimal cutoff value of plasma IL-34 yielding 87.1% sensitivity and 100.0% specificity. Since advances in soluble biomarkers directly linked to synovitis pathology may well yield diagnostic tools for appropriate stratification in early phases of synovitis, IL-34 produced by knee OA FLS and released into the circulation and joint fluid of knee OA would be an additional inflammatory molecule involved in the development and progression of synovitis and cloud serve as a potential biomarker for knee OA synovitis. It is important to note that targeting the synovium to counteract IL-34 overproduction and to subsequently halt the excess accumulation of matrix-degrading mediators would be of great clinical relevance. In view of our considerable findings presented herein, it is tempting to assume that augmented transcriptional and translational expressions of IL-34 in the systemic and local joint environments of knee OA patients, particularly those with synovitis may result from compensation mechanisms by the body in response to an imbalance between the anabolic and catabolic processes in the joint contributing to synovial inflammation and subsequent cartilage destruction. This phenomenon could intensify the release of IL-34 from the specific local tissues like the inflamed synovium into the synovial fluid and circulation, which has been attested by our results revealing overexpressions of IL-34 mRNA and protein in both *in vitro* and *in vivo* models. For no statistically significant differences in synovial fluid IL-34 levels among knee OA with different degrees of synovitis, the possible explanation may be attributed to the source of IL-34 in the synovial fluid that has been reportedly originated from the articular cartilage^11^, aside from FLS and the inflamed synovium. Given its additional role in osteoclastogenesis^7^, increased secretion of IL-34 from the articular cartilage into synovial fluid may result from cartilage degradation in knee OA patients. This speculated fate may help explain why we did not find statistically significant difference in synovial fluid IL-34 levels between the patients with and without synovitis who both suffer from cartilage destruction; however, there was a tendency of increased IL-34 levels in synovial fluid of knee OA patients with regard to synovitis severity.

Despite substantial findings demonstrated in this study, we should be aware of some inherent limitations. Firstly, the causal associations of IL-34 mRNA and protein expressions with the severity of knee OA synovitis were not fully addressed in the study, because of the cross-sectional design with relatively small numbers of study participants. Regarding this, multi-center prospective cohorts with larger sample sizes are required to evaluate whether IL-34 overexpression is causally related to the progressive knee OA synovitis or whether it is simply a defensive response to the disease. Another caveat is lack of patient data on physical performance tests and clinical symptoms, in which we were unable to determine whether aberrant IL-34 expression is correlated with functional impairment and the severity of knee pain. It is recommended that future studies should collect prospective measurements of these data to preclude bias and reverse causation. Additionally, due to ethical considerations, harvesting the synovium and joint fluid specimens from healthy volunteers were limited. In future investigations, this may be overwhelmed by using patients with non-arthritic knee surgery as controls.

Taken together, the present study provides supporting evidence of alterations in IL-34 expression regulated by TNF-α in knee OA FLS. Furthermore, transcriptional and translational levels of IL-34 were significantly elevated in knee OA synovium with severe synovitis, compared with those with mild and no synovitis. Subsequent analysis disclosed increases in synovial fluid and plasma IL-34 levels in knee OA patients with severe synovitis. Additionally, IL-34 expression and production in the systemic and local joint environments were found to be positively associated with the severity of knee OA synovitis. Altogether, plasma IL-34 appears to have potential as a non-invasive biomarker indicating the development and progression of knee OA synovitis. Further investigations are still essential for better understanding of IL-34 exact role in the pathogenesis of knee OA synovitis, which would open novel therapeutic opportunities.

## Materials and Methods

### Patients and synovial biopsies

The study protocol conformed to the guidelines of the Declaration of Helsinki and was approved by the Institutional Review Board of the Faculty of Medicine, Chulalongkorn University (IRB number 533/54) and the Faculty of Dentistry/Faculty of Pharmacy, Mahidol University (IRB number 2018/072.1812). All participants were fully informed regarding the study protocol and procedures prior to their entering the study. Written informed consent was obtained from the participants.

Synovial biopsies of 50 knee OA patients were harvested surgically at the time of an arthroplasty. All knee OA subjects recruited in the current work were diagnosed with knee OA in accordance with the criteria of the American College of Rheumatology (ACR) based on clinical and radiographic classification^24^. All the knee OA patients had radiographic findings determined by anteroposterior weight-bearing radiographs of the affected knees in extension. Samples of synovium were collected from the knees of 12 OA patients at the time of diagnostic or therapeutic arthroscopy, and all of them had no synovitis or minor synovitis. Synovial samples of 38 knee OA patients were obtained at the time of total joint arthroplasty. After macroscopic evaluation, normal synovium or no synovitis were defined as few translucent areas and slender villi with a fine vascular network, whereas inflamed synovium or synovitis were characterized as hypervascularized areas with hypertrophic and hyperemic villi. None of the participants had underlying diseases such as diabetes mellitus, advanced liver or renal diseases, histories of long-term steroid treatment, other forms of arthritis, previous knee injury and/or infection, malignancy, or other chronic inflammatory diseases.

### Cell isolation and culture

As an excellent cellular model for studying the pathological physiology of synoviocytes and the eventual development of joint diseases, FLS was isolated from synovial tissues of 4 knee OA patients who underwent TKR using enzymatic digestion, according to standard protocols. In brief, synovium was sectioned into small pieces using a Bard Parker blade under sterile conditions. The tissue fragments were combined with sterile collagenase solution (Sigma, St. Louis, MO, USA). The mixture was agitated at 37 °C, 5% CO_2_, and 95% humidity for 6 h. After collagenase digestion, cells were recovered through centrifugation to yield a cellular pellet that was re-suspended in Dulbecco’s modified Eagle medium (DMEM, Hyclone Laboratories Inc., South Logan, UT) supplemented with 100 IU/mL penicillin/streptomycin (Hyclone Laboratories Inc., South Logan, UT) and 10% fetal bovine serum (Hyclone Laboratories Inc., South Logan, UT). The cell solution was transferred to a 75 cm^2^ tissue culture flask (Nunc, Roskilde, Denmark) containing 10 mL of supplemented DMEM. The flasks were incubated at 37 °C in a humidified atmosphere of 5% CO_2_, with sterile medium change performed every 3 days until the cells reached 95% confluence. Cells at the first passage (P1) were used for the experiments.

In experimental groups, FLS was treated with and without TNF-α for 1, 3, and 7 days at 37 °C and 5% CO_2_ in a humidified environment. The treatment was carried out in serum-free medium, and the concentration of 10 ng/mL TNF-α (Biolegend, San Diego, CA, USA) was selected, according to previously published studies^8,25,26^.

### Quantitative real-time polymerase chain reaction (PCR)

Relative mRNA expressions of *IL-34* and signaling molecules involved in NF-κB pathway including *IL-6*, *IκB, NF-κB*, and *MMP-13* were investigated using quantitative real-time PCR. Total RNA was extracted from synovial tissues of knee OA patients and knee OA FLS stimulated with and without TNF-α using RNeasy Mini Kit (Qiagen, Hilden, Germany), with cDNA reverse transcribed using TaqMan Reverse Transcription Reagents (Applied Biosystems, Inc., Foster City, CA, USA). Real-time PCR was performed using QPCR Green Master Mix HRox (biotechrabbit GmbH, Hennigsdorf, Germany) on StepOnePlus Real-Time PCR System (Applied Biosystems, Inc., Foster City, CA, USA). Primers used for *IL-34*, *IL-6, IκB, NF-κB, MMP-13*, and glyceraldehyde 3-phosphate dehydrogenase (*GADPH*) amplifications are demonstrated in Supplementary table 1. Relative mRNA expressions of *IL-34, IκB, NF-κB, IL-6*, and *MMP-13* normalized to *GADPH* as an internal control were determined using 2^−∆∆Ct^ method, as previously described by Udomsinprasert *et al*.^27^.

### Hematoxylin and eosin (H&E) and immunohistochemistry

Localization of IL-34 protein expression in synovial tissues of knee OA patients was determined using immunohistochemical analysis. The tissue specimens were paraffin-embedded and subsequently sectioned, according to standard protocols. Routine staining with hematoxylin and eosin (H&E) and immunohistochemistry staining with antibodies was conducted to determine morphological changes in the synovium and detect IL-34 protein expression (Abcam, Cambridge, MA, USA). A standard immunohistochemical technique was performed using an autostainer (Ventana Medical Systems Inc., Tucson, AZ, USA). Briefly, tissue sections were deparaffinized and rehydrated. Endogenous peroxidase activity was blocked by 0.3% hydrogen peroxide for 10 min. Following heat-induced antigen retrieval in 10 mmol/L citrate buffer (pH 6) for 5 min, the slides were incubated in pepsin for 7 min and subsequently incubated with 1:500 diluted primary antibodies for 2 h. Afterwards, the sections were stained with the secondary antibody conjugated to streptavidin/horseradish peroxidase for 45 min at room temperature. Reaction products were visualized using 3,3-diaminobenzidine tetrahydrochloride (Sigma, St. Louis, MO, USA), and the sections were counterstained with hematoxylin, as previously delineated^26^.

A histopathological classification system was used to determine the severity of synovial inflammation. This synovitis score is estimated using conventionally stained routine sections with H&E and generally based on the following relevant morphological alterations: hyperplasia/enlargement of synovial lining cell layer, activation of resident cells/synovial stroma, and infiltration of inflammatory cells. All defined histopathological qualities are graded from absent (0 point), slight (1 point), and moderate (2 points) to strong (3 points), in which three averages were summed to create a total score of synovitis ranging from 0 to 9. According to the synovitis score, the histological findings are generally classified into three groups: no synovitis (0–1 point), low-grade synovitis (2–4 points), and high-grade synovitis (5–9 points), as previously described^28^.

For IL-34 immunohistochemical staining, cells with brown stained cytoplasm were scored as positive. All tissue sections were analyzed by a pathologist who was blinded to clinical status and diagnosis of the patients. The immunoreactivity of IL-34 was semi-quantitatively analyzed for percentage of positive cells and intensity of staining. A percentage of positive cells <1% was scored as 0; 1–25% as 1; >25–50% as 2; and >50% as 3. The intensity of IL-34 immunostaining was determined using the following staining scores: 0 = no staining; 1 = weak staining; 2 = moderate staining; and 3 = strong staining. Final results were scored by the total score [total scores = ((score of positive cell × score of intensity) × 100)/maximum score of both parameters]. Using the aforementioned staining scores, the positive areas of positive cells of IL-34 were determined by measuring five randomly selected microscopic fields (200×) on each slide, following our previous study^27^.

### Enzyme-linked immunosorbent assay (ELISA)

Venous fasting blood samples were drawn from knee OA patients in ethylenediaminetetraacetic acid, centrifuged, and stored immediately at −20 °C for later measurement. Synovial fluid was aspirated from the OA patients using sterile knee puncture just prior to surgery when the arthroscopy or total knee arthroplasty were performed. The specimen was subsequently centrifuged to remove cells and joint debris and stored at −20 °C until analysis. Plasma and synovial fluid IL-34 levels were quantitatively determined using a commercial sandwich enzyme-linked immunosorbent assay (ELISA) kit (R&D Systems, Minneapolis, MN, USA), according to the manufacturer’s instructions^29^.

### Statistical analysis

All statistical analyses were performed using the statistical package for social sciences version 22.0 (SPSS, Inc., Chicago, IL, USA). Demographic and clinical characteristics among groups were evaluated using Chi-square tests and one-way analysis of variance where appropriate. Comparisons among each group were executed by Mann-Whitney *U*-test (for 2 groups) or Kruskal-Wallis *H* test (for > 2 groups). Comparisons in mRNA expressions in knee OA FLS at intervals of 1, 3, and 7 days between groups were accomplished by Wilcoxon signed-rank test. Correlations of *IL-34* mRNA expression with its protein expression, mRNA expressions of *NF-κB* and its signaling molecules, and the severity of synovitis were assessed using Spearman’s rho correlation coefficient (*r*). Multivariate linear regression models were conducted to determine the roles of confounding factors. Receiver operating characteristic (ROC) curve and the area under the ROC curve (AUC) were calculated to assess the feasibility of using plasma IL-34 as a possible biomarker for knee OA synovitis. All data are represented as a mean ± standard deviation (SD). A two-tailed *P*-value of less than 0.05 was considered statistically significant for all analyses.

## Supplementary information

Supplementary Information.
