# Supplementary material for: Interleukin-34 overexpression mediated through tumor necrosis factor-alpha reflects severity of synovitis in knee osteoarthritis
Source: Sci Rep. 2020 May 14;10:7987. doi: 10.1038/s41598-020-64932-2 (PMC7224362; doi:10.1038/s41598-020-64932-2)
Supplement: Supplementary file 1 — Supplementary Information. [file 41598_2020_64932_MOESM1_ESM.docx]

**Supplementary table 1** Primers used for mRNA expression analyses.

| **Genes** | **Primers** | **Sequence** |
| --- | --- | --- |
| ***IL-34*** | Forward | 5’-GTGCTTAGGCCTCTGTGGAC-3’ |
|  | Reverse | 5’-GCCAAGGAAGATCCCAAGATA-3’ |
| ***IL-6*** | Forward | 5′-AGCCACTCACCTCTTCAGAAC-3′ |
|  | Reverse | 5′-ACATGTCTCCTTTCTCAGGGC-3′ |
| ***IκB*** | Forward | 5′-GGCTGAAGAAGGAGCGGCTA-3′ |
|  | Reverse | 5′-CCATCTGCTCGTACTCCTCG-3 |
| ***NF-κB*** | Forward | 5′-GACCGCTGCATCCACAGTTT-3′ |
|  | Reverse | 5′-GGATGCGCTGACTGATAGCC-3′ |
| ***MMP-13*** | Forward | 5′-TTGCAGAGCGCTACCTGAGATCAT-3′ |
|  | Reverse | 5′-TTTGCCAGTCACCTCTAAGCCGAA-3′ |
| ***GAPDH*** | Forward | 5’-GTGAAGGTCGGAGTCAACGG-3’ |
|  | Reverse | 5’-TCAATGAAGGGGTCATTGATGG-3’ |

Abbreviations: GAPDH, glyceraldehyde 3-phosphate dehydrogenase; IκB, I kappa B protein; IL-34, interleukin-34; IL-6, interleukin-6; MMP-13; matrix metallopeptidase-13; NF-κB; nuclear factor kappa B.
